# Covalent grafting of molecular catalysts on C_3_N_*x*_H_*y*_ as robust, efficient and well-defined photocatalysts for solar fuel synthesis†

**DOI:** 10.1039/d0sc02986f

**Published:** 2020-07-24

**Authors:** Christopher D. Windle, Alexander Wieczorek, Lunqiao Xiong, Michael Sachs, Carlota Bozal-Ginesta, Hyojung Cha, Jeremy K. Cockcroft, James Durrant, Junwang Tang

**Affiliations:** Department of Chemical Engineering UCL Torrington Place London WC1E 7JE UK junwang.tang@ucl.ac.uk; Department of Chemistry and Centre for Processable Electronics, Imperial College London, White City Campus London W12 0BZ UK; Department of Chemistry, University College London 20 Gordon Street London WC1H 0AJ UK

## Abstract

The covalent attachment of molecules to 2D materials is an emerging area as strong covalent chemistry offers new hybrid properties and greater mechanical stability compared with nanoparticles. A nickel bis-aminothiophenol catalyst was grafted onto a range of 2D carbon nitrides (C_3_N_*x*_H_*y*_) to form noble metal-free photocatalysts for H_2_ production. The hybrids produce H_2_ beyond 8 days with turnover numbers reaching 1360 based on nickel, a more than 3 fold higher durability than reported molecular catalyst-carbon nitride mixtures, and under longer wavelengths (>475 nm). Time-resolved spectroscopy reveals sub-microsecond electron transfer to the grafted catalyst, six orders of magnitude faster compared with similar reports of non-grafted catalysts. The photoelectrons on the catalyst have a *ca.* 1000 times longer half-time (7 ms) compared with bare carbon nitride (10 μs). The grafting strategy operates across a range of molecular catalyst-carbon nitride combinations, thus paving the way for robust efficient photocatalysts based on low-cost tunable components.

## Abstract 2

A nickel bis-aminothiophenol catalyst grafted onto a 2D carbon-nitride photocatalytically produces H_2_ beyond 8 days with turnover numbers reaching 1360.

## Graphical abstract

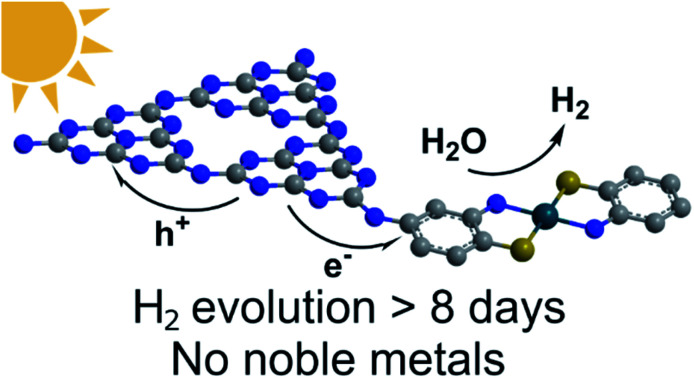

## Introduction

Photocatalytic water splitting has the potential to provide pollution-free fuel, thus combatting climate change and poor air quality. The so-called Z-scheme is one promising route to overall water splitting with sunlight. This biomimetic approach uses two photocatalysts and offers a higher theoretical efficiency than one.^1^ However, several challenges must be overcome to realize a commercially-viable solution and these rest on the development of new photocatalysts for each half reaction: (i) hydrogen production must be cost-effective but most examples utilize rare expensive metals (Pt, Rh) (ii) the hydrogen evolution catalyst must be selective to prevent efficiency loss *via* back reactions (iii) light-absorption by the photocatalysts must be tunable to cover large and complementary areas of the visible spectrum (iv) the photocatalysts must be durable.

The combination of carbon nitride with molecular catalysts is a promising solution to these challenges. Carbon nitride is a low-cost photocatalyst comprised of carbon, nitrogen and hydrogen in an ideal stoichiometry of C_3_N_4_. In reality the stoichiometry deviates from the ideal, expressed as C_3_N_*x*_H_*y*_ and herein denoted CN.^2^ The absorption profile of CN can be tuned to produce hydrogen at wavelengths as long as 700 nm.^3^ While most reports of CN utilize Pt as a hydrogen evolution catalyst,^3–5^ there are a number of molecular catalysts based on Earth-abundant elements that could be utilized though scarcely reported.^6^ Molecular catalysts can also offer good selectivity for hydrogen evolution over oxygen reduction.^7^ This challenge is critical for Z-schemes and is one that Pt alone cannot overcome. There are a few scarce examples of molecular catalysts for hydrogen evolution combined with CN.^8–11^ The reported systems are limited by poor durability, caused by the instability of the catalyst and the instability of the interaction between the catalyst and CN. The longest reported running time is 60 h^10^ and the highest reported turnover number (TON) is 425.^9^ In these systems the catalysts are freely diffusing in solution with only a tiny fraction of molecules interacting with the CN surface. In one case washing the CN particles led to a 92% drop in activity, highlighting that phosphonate groups do not form a strong interaction with CN.^8^ It is clear that strong covalent chemistry between molecular catalysts and CN is an important target. In addition, covalent attachment to CN improves the recyclability of the molecular catalyst and offers greater mechanical stability compared with nanoparticle catalysts. Mechanical stability is important if further processing is required to prepare higher-order composites (*e.g.* solid Z-schemes) *via* high-energy techniques such as ultra-sonication and ball milling. Despite the abundant amine groups on the surface of CN there are only few examples of a well-defined molecular catalyst covalently bound to the surface, suggesting that activation of the amine groups is challenging. Interestingly one can see there is not such catalyst used for water splitting while there are few reports on CO_2_ reduction.^12,13^ In some other examples, CN was functionalized with thiol groups and NiCl_2_ was absorbed but the resulting catalyst structure is poorly defined.^14^ In another example porphyrins and phthalocyanines were bound to CN as photosensitizers but not as catalysts.^15,16^

The covalent attachment of molecules to 2D materials is an emerging area. The resulting hybrids have exhibited remarkable new properties in the field of electrochemistry. The new materials behave more like catalytically active metal surfaces than molecules and show excellent durability (TON 12,000).^17–19^ To the best of our knowledge no photocatalytic examples for hydrogen evolution have been reported at present.

We selected a nickel bis-aminothiophenol catalyst because it is robust in solution (TON 293,000) and highly selective (78% faradaic efficiency for H_2_ in air).^20^ We prepared a novel catalyst with chloride groups allowing for its covalent attachment to a range of doped and non-doped CNs. The hybrids show efficient hydrogen evolution for more than 8 days, fast (<μs) and long-lived (ms) charge separation and activity under *λ* > 475 nm.

## Results and discussion

### Synthesis and characterization

**Fig. 1:**
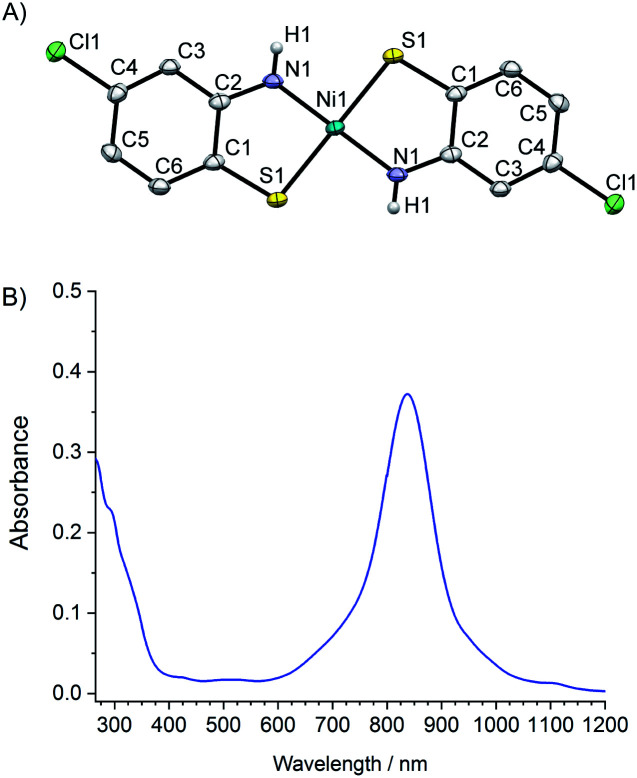
(A) X-ray crystal structure of **Ni(abtCl)2**, thermal ellipsoids shown at 50% probability (B) UV/Vis absorption spectra of **Ni(abtCl)2** at 10^−5^ M in DMF.

**Ni(abtCl)2** (Fig. 1A) was prepared using a modification of a literature procedure to coordinate 2-amino-4-chlorobenzenethiol to nickel. The structure and purity were confirmed by single crystal X-ray diffraction and elemental analysis, respectively. Single crystals suitable for X-ray diffraction were grown by slow diffusion of CHCl_3_ into DMSO (Fig. 1A). The Ni is square planar with a S–Ni–N angle of 88°, identical to the non-chlorinated analogue.^20^ The UV/Vis spectrum of **Ni(abtCl)2** in DMF solution (Fig. 1B) exhibits a strong absorption band at 838 nm assignable to a ligand-to-ligand charge transfer (LLCT) band. It shows a red shift compared with the corresponding complex without chloride groups (815 nm).^20^

### Preparation and characterization of hybrid materials

**Scheme 1:**
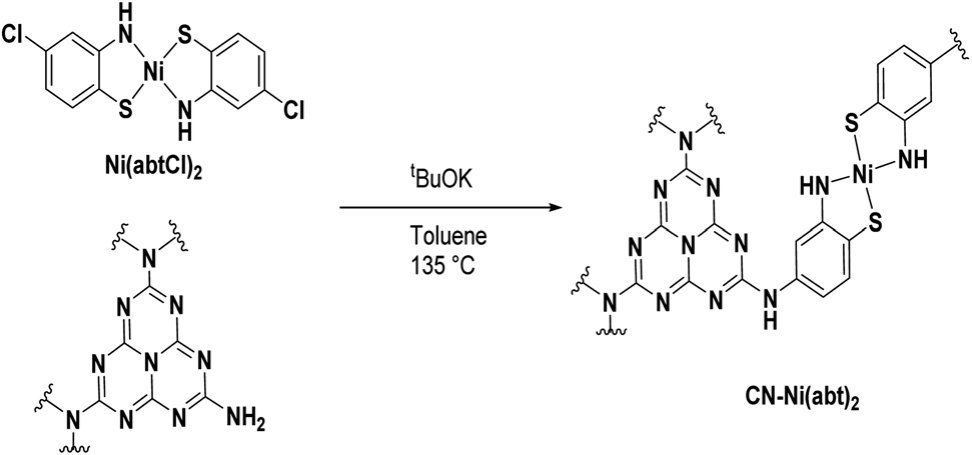
Grafting of molecular catalysts onto CN.

The hybrid materials were prepared using a coupling reaction between aryl amines and aryl chlorides catalyzed by potassium *tert*-butoxide (Scheme 1).^21^ In a pressure tube, CN, **Ni(abtCl)2** and ^*t*^BuOK were suspended in toluene and heated to 135 °C for 36 h. The reaction was quenched with water and the solid was thoroughly washed by sonication in DMSO until the washings were colorless to remove any adsorbed material. The solid was finally washed with water and dried at 70 °C overnight. Hybrids were prepared of **Ni(abtCl)2** with four different CNs. We used CN derived from urea^5^ and from dicyandiamde (DCDA), and two oxygen-doped CNs; formic acid treated (FAT)^4^ and oxygen nitrogen linked heptazine (ONLH).^3^ They are denoted CN_urea_-**Ni(abt)2**, CN_DCDA_-**Ni(abt)2**, FAT-**Ni(abt)2** and ONLH-**Ni(abt)2**.

**Fig. 2:**
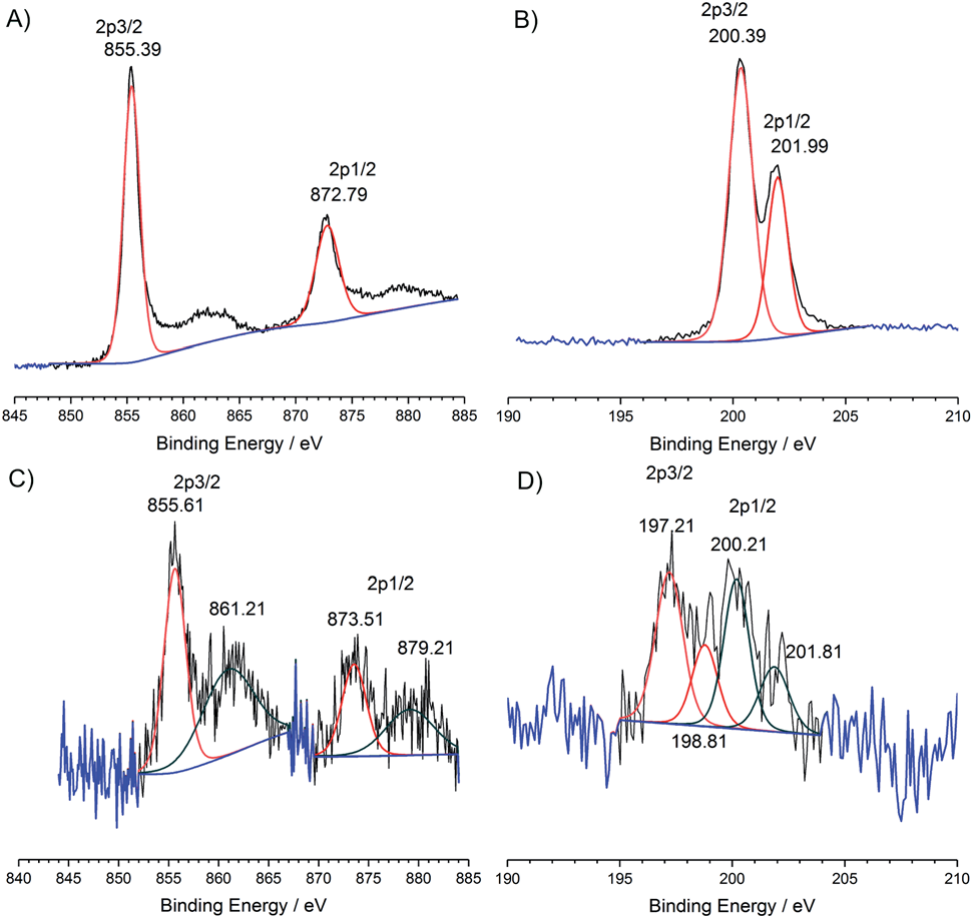
X-ray photoelectron spectra of pure **Ni(abtCl)2**, (A) Ni 2p region (B) Cl 2p region and CN_urea_-**Ni(abt)2** hybrid (C) Ni 2p region (D) Cl 2p region.

The CN_urea_-**Ni(abt)2** hybrid was characterized using X-ray photoelectron spectroscopy (XPS). Pure **Ni(abtCl)2** exhibits Ni 2p signals at 855.39 and 872.79 eV assignable to Ni^II^ (Fig. 2A).^22^ The typical Ni satellite signals are present.^23^ Chloride 2p signals are observed at 200.39 and 201.99 eV assignable to organic chloride (Fig. 2B).^24^ The ratio of atomic abundance between Ni and Cl is 1 : 2, in good agreement with the molecular structure. The hybrid material CN_urea_-**Ni(abt)2** displays clear signals for nickel and chloride. Ni 2p signals are observed at 855.61 and 873.51 eV in close agreement with the pure molecule (Fig. 2C). The satellite signals are retained although their intensity relative to the parent is increased. This may be due to the complex origins of satellite signals in Ni X-ray photoelectron spectra^23^ or there may also be a contribution from a trace of Ni^III^. The chloride signal is very weak (Fig. 2D) due to a dramatic loss in chloride content from the coupling reaction with the amine groups of the CN as illustrated in Scheme 1. The chloride signal fits to four peaks, indicating the presence of two different chloride 2p environments with binding energies corresponding to organic chloride and inorganic chloride.^25^ The presence of inorganic chloride suggests some chloride released during the coupling reaction coordinates to the Ni center and this is consistent with a trace of Ni^III^. The ratio of atomic abundance between Ni, Cl_organic_ and Cl_inorganic_ is 1 : 0.25 : 0.28, indicating that there has been an 88% loss in chloride bound to the aminothiophenol ring, thus confirming that the coupling reaction has taken place.

**Fig. 3:**
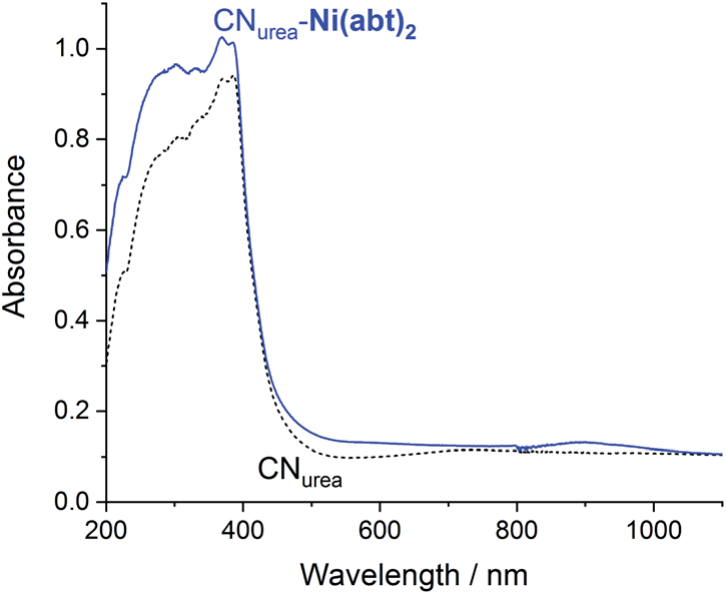
Diffuse reflectance UV/Vis spectra of CN_urea_ and CN_urea_-**Ni(abt)2**.

The CN_urea_-**Ni(abt)2** hybrid material was then characterized using diffuse reflectance UV/Vis spectroscopy (DRUVS) (Fig. 3). Comparison of CN_urea_-**Ni(abt)2** with bare CN_urea_ shows a new absorption maximum at 896 nm and an enhanced absorption throughout the UV/Vis region, originating from the presence of **Ni(abt)2** on the surface. Retention of the LLCT band is a clear indication that the molecular structure is retained after the reaction with CN. The long-wavelength absorption maximum of **Ni(abt)2** bound to CN is red shifted by 58 nm compared with **Ni(abtCl)2** in DMF solution.

The quantity of **Ni(abt)2** bound to CN was measured *via* inductively coupled plasma atomic emission spectroscopy (ICP-AES) to be 0.23 μmol per 10 mg CN_urea_. This equates to a 0.1 wt% loading of Ni. The catalyst loadings for each hybrid are given in Table 1, along with the surface areas of the materials. There is some correlation between surface area and catalyst loading; however, CN_DCDA_ shows the lowest loading despite having a higher surface area than FAT, indicating that surface chemistry and not just surface area is important for catalyst loading.

**Table 1:** Catalyst loadings in the hybrids and surface areas for each material

Hybrid	**Ni(abt)2** loading/μmol per 10 mg	Surface area/m^2^ g^−1^
CN_DCDA_-**Ni(abt)2**	0.054	13.8 (ref. 3)
FAT-**Ni(abt)2**	0.095	12.1 (ref. 4)
ONLH-**Ni(abt)2**	0.168	32.9 (ref. 3)
CN_urea_-**Ni(abt)2**	0.233	43.8 (ref. 5)

### Photocatalytic activity

**Fig. 4:**
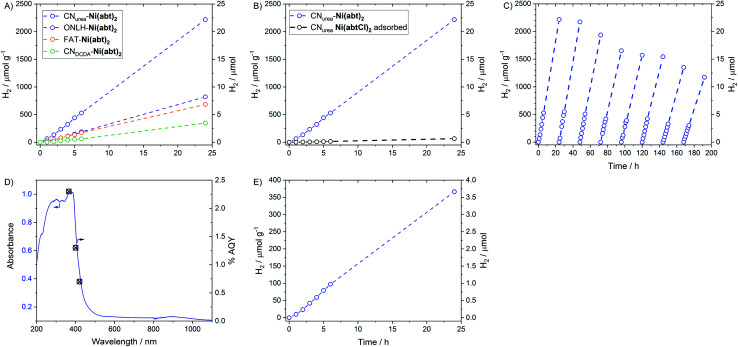
Photocatalytic hydrogen production by (A) CN-**Ni(abt)2** hybrids (B) CN_urea_-**Ni(abt)2** compared with CN_urea_ with **Ni(abtCl)2** absorbed (C) CN_urea_-**Ni(abt)2** activity for 8 days (D) quantum yields of H_2_ overlaid with DRUVS spectrum for CN_urea_-**Ni(abt)2**. All in H_2_O with 10% TEOA and *λ* > 420 nm (pH = 11). (E) ONLH-**Ni(abt)2** with *λ* > 475 nm.

The CN-**Ni(abt)2** hybrids were tested for photocatalytic H_2_ production with TEOA as a widely used electron donor for polymer photocatalysts under visible light (*λ* > 420 nm, 300 W Xe lamp). All four hybrids display H_2_ evolution with linear activity over 24 h, indicating excellent stability (Fig. 4A). The result also confirms the versatility of the grafting procedure across a range of CNs. The most efficient is CN_urea_-**Ni(abt)2** followed by ONLH-**Ni(abt)2**, then FAT-**Ni(abt)2** and finally CN_DCDA_-**Ni(abt)2**. The high efficiency of the CN_urea_ hybrid with a H_2_ evolution rate of *ca.* 100 μmol g^−1^ h^−1^ is unsurprising as urea-derived CN is known to be highly efficient with a range of co-catalysts and for a range of reactions^5,26^ as well as possessing a very high quantum yield in the presence of Pt co-catalyst. This is due to the faster charge transfer in the sample with a higher degree of polymerisation.^5^ The trend observed for the other three materials correlates well with their abilities to harvest visible light. The amount of catalyst loading has a positive correlation with the catalytic activity, emphasising the effectiveness of the molecular catalyst. The amount of catalyst loaded is dominated by the surface area as indicated in Table 1 while other factors (*e.g.* degree of protonation or other surface state) also play a role as indicated by the sample CN_DCDA_ which shows the lowest loading amount despite with a higher surface area than FAT. The activity is found to be dependent on the pH value of the solution (Fig. S2†). The activity decreases with decreasing pH due to the electrostatic repulsion of protonated, positively charged TEOA.^27^ We next loaded similar amount of Pt on CN_urea_ by photodeposition, and compared its activity with CN_urea_-**Ni(abt)2**. It is shown that the H_2_ evolution rate and turnover frequency of CN_urea_-**Ni(abt)2** are 0.92 μmol h^−1^ and 9.2 h^−1^ respectively, while those of Pt loaded CN are 1.55 μmol h^−1^ and 6.9 h^−1^ under the same condition (Fig. S3†), proving the comparative effectiveness of the new molecular catalyst to the well know benchmark catalyst Pt. To test the importance of covalent binding **Ni(abt)2** to the CN surface, **Ni(abtCl)2** was adsorbed onto CN_urea_ from a DMF solution and then worked-up in the same way as for CN_urea_-**Ni(abt)2**. When **Ni(abtCl)2** is absorbed onto CN_urea_ only a trace of H_2_ is produced (Fig. 4B). The CN_urea_-**Ni(abt)2** hybrid was tested for 192 h (8 days) over which time the activity dropped by just 50%. This is a very significant improvement over other reported molecular catalyst-semiconductor hybrids, which typically deactivate within hours (Table S2†). To further clarify the deactivation, the solution was analysed after 4 days irradiation (*λ* > 420 nm) when the activity dropped by 25%, the quantity of Ni^II^ in the solution was measured *via* ICP-AES to be 0.06 μmol in 50 mL of H_2_O, indicating that there was a 26% loss of Ni atoms from the molecular catalyst to the solution. This means the loss of activity is mainly derived from the photodecomposition of the molecular catalyst, which is a general problem encountered in molecular catalysts.^8,28^ A total of 136 μmol of H_2_ were produced during this time. The quantity of **Ni(abt)2** on the CN_urea_ was measured again after 24 h photocatalysis by ICP-AES as 0.1 μmol of **Ni(abt)2** per 10 mg CN_urea_. This results in a TON with respect to **Ni(abt)2** of 1360. To provide a measure of the photocatalyst efficiency independent of the lamp intensity the quantum yield for H_2_ of CN_urea_-**Ni(abt)2** was measured for a range of wavelengths (Fig. 4D) and reaches *ca.* 1.5% in the visible region (400 nm).

To obtain a more accurate picture of the intrinsic capability of the grafted catalyst, irradiation with high-energy UV light was investigated. Under these conditions more photoelectrons should be available to the catalyst, albeit the system is less stable. The rate of H_2_ production is much higher with *λ* > 320 nm (Fig. S4†) and gives a TOF = 54.3 h^−1^ in the first hour, nine times greater than under *λ* > 420 nm. The activity then degrades due to UV damage to the molecular catalyst as proved above. This provides further evidence that the active species is the molecular catalyst, as decomposition of the catalyst leads to deactivation and not to the formation of a catalytically active material.

A major advantage of the oxygen-doped CNs over their non-doped counterparts is their ability to efficiently utilize a wider range of the visible spectrum. Under *λ* > 475 nm ONLH-**Ni(abt)2** stably and efficiently produced H_2_ (Fig. 4E).

### Identification of the active species

After 24 h irradiation (*λ* > 420 nm) the CN_urea_-**Ni(abt)2** was separated from the solution and analyzed by XPS in the Ni 2p region (Fig. S1†). Clear Ni signals confirm that the catalyst is retained on the CN surface. There is no change in the binding energy for the Ni signals compared with the freshly prepared hybrid material CN_urea_-**Ni(abt)2** and compared with the pure molecule (Fig. 2), indicating that the catalyst structure is retained and there is not Ni metallic species observed. In addition, in order to confirm the superior activity of CN_urea_-**Ni(abt)2**, we loaded similar amount of NiO on CN_urea_ by an impregnation method. It turned out that CN_urea_-NiO is much less active than CN_urea_-**Ni(abt)2**, *e.g.* at least one order of magnitude worse (Fig. S5†). Based on these results, one can say that the cocatalyst structure is retained after long term reactions, and neither Ni metallic species nor NiO as a cocatalyst could achieve similar activity to the bonded molecular **Ni(abt)2**.

### Versatility of the method

**Fig. 5:**
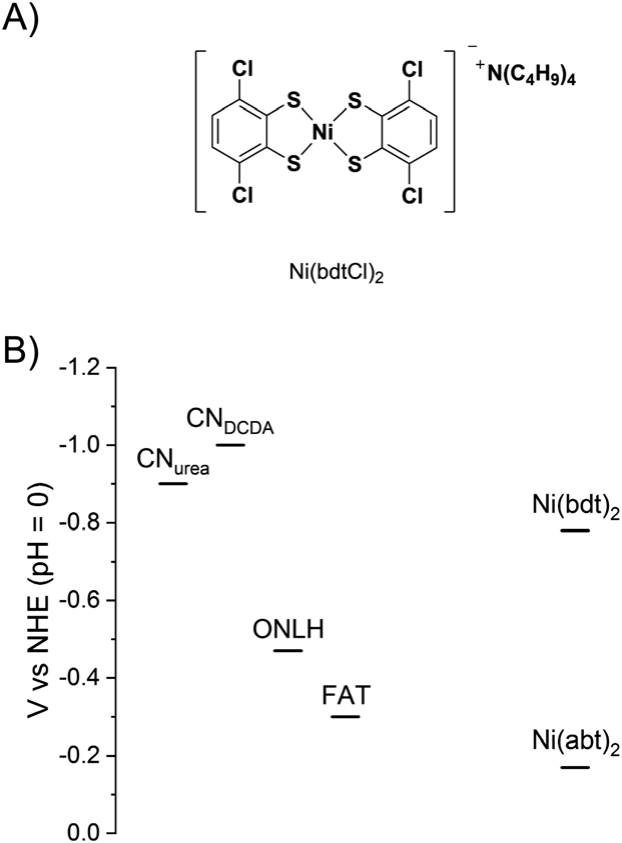
(A) Structure of **Ni(bdtCl)2** (B) estimated potentials of the CN conduction bands and the onset of H_2_ evolution for **Ni(bdt)2** and **Ni(abt)2**.^3,4,20,26^

To test the versatility of the covalent bonding approach and to provide further evidence that the molecular structure is retained in the hybrid materials, a second catalyst was bound to CN. The structure of **Ni(bdtCl)2** is shown in Fig. 5A. The Ni benzene dithiol catalyst core has an overpotential for H_2_ evolution that is 610 mV more negative than **Ni(abt)2**.^20^ The hybrid materials based on **Ni(bdt)2** also produce H_2_ under the same conditions as used for the **Ni(abt)2** hybrids (Fig. S6 and Table S3†). However, the activity is lower and the hybrids with ONLH and FAT are inactive. The conduction band potentials of the CNs and the potentials for the onset of H_2_ evolution of **Ni(abt)2** and **Ni(bdt)2** are given in Fig. 5B. It is clear that only CN_urea_ and CN_DCDA_ are sufficiently reducing to transfer electrons to both catalysts. ONLH and FAT cannot transfer electrons to **Ni(bdt)2**. On the other hand there is a significantly greater driving force for electron transfer from CN_urea_ and CN_DCDA_ to **Ni(abt)2** than to **Ni(bdt)2**, which can explain the higher rates of H_2_ evolution for the **Ni(abt)2** hybrids. The reduction in activity when using a less efficient molecular catalyst strongly suggests that the molecular structure is retained on the CN surface. In the case of the **Ni(abt)2** hybrids the CN_DCDA_ is less active than the ONLH and FAT despite having a more negative conduction band and this is attributed to the greater visible light harvesting ability of ONLH and FAT. The overall superior performance of CN_urea_ is consistent with the very high quantum yield compared with other forms of CN.^5^

### Transient absorption spectroscopy

To understand how photogenerated charges are being transferred from CN to the covalently bound molecular catalyst, CN_urea_ and CN_urea_-**Ni(abt)2** were investigated *via* transient absorption spectroscopy (TAS) on the μs to s timescale. In previous kinetic studies, the transfer of photogenerated charges to the co-catalyst was found to compete with deep charge trapping in analogous CN.^28^ Deep trapping of electrons was found to significantly lower the hydrogen evolution rates in the studied types of CN.^28^ Therefore, faster transfer of charges from CN to the co-catalyst may lead to significant improvements in the overall performance of the system.^29^

**Fig. 6:**
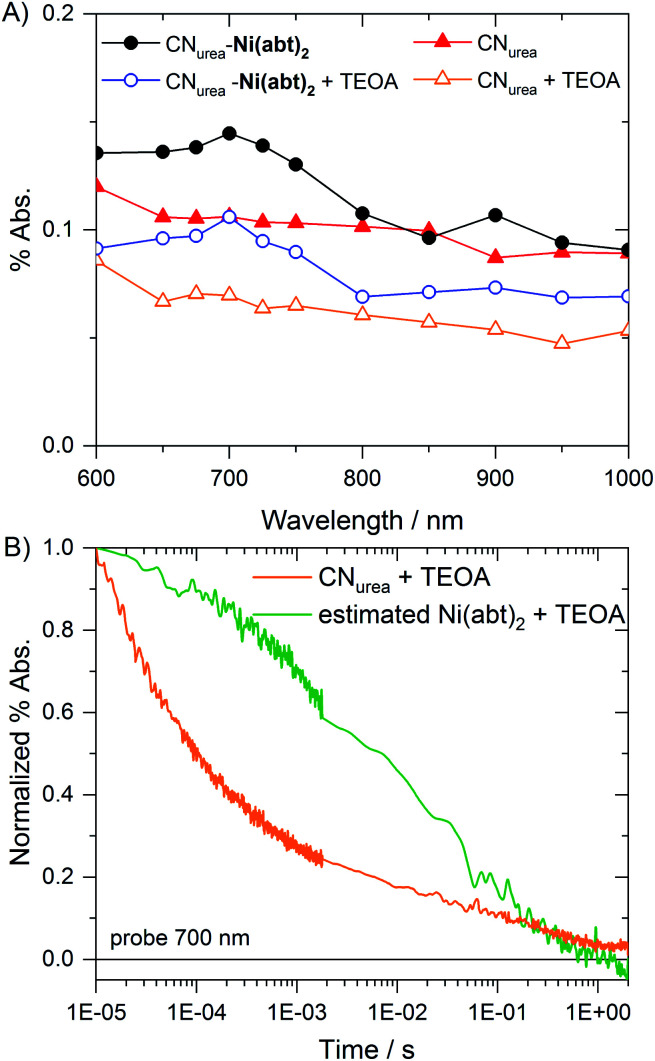
Temporal evolution of photogenerated reaction intermediates. (A) Transient absorption spectrum of CN_urea_ and CN_urea_-**Ni(abt)2** at 100 μs in pure water and in an aqueous 10 vol% TEOA solution. (B) Normalized kinetics of CN_urea_ in TEOA at 700 nm with estimated kinetics from the transfer of charges from carbon-nitride to the co-catalyst. The estimated kinetics were obtained by subtraction of the CN_urea_ from CN_urea_-**Ni(abt)2** kinetics in TEOA and followed normalization. Probed at 700 nm for 355 nm excitation density of 500 μJ cm^−2^.

Samples were prepared by suspending CN_urea_ and CN_urea_-**Ni(abt)2** in water and further diluting to obtain suspensions in pure water and with added 10 vol% TEOA. Upon photoexcitation at 355 nm, a broad featureless absorption signal from 600–1000 nm is observed for CN_urea_ (Fig. 6A). This signal was previously attributed to deeply trapped charges which decay *via* bimolecular recombination in the absence of a cocatalyst.^28^ For CN_urea_-**Ni(abt)2**, an additional peak-like spectral feature is found in the 600 to 800 nm region, both in the presence and absence of TEOA. Since this additional feature is only observed in CN_urea_-**Ni(abt)2**, it is herein assigned to electrons at least partially located on the **Ni(abt)2** co-catalyst.

The decay kinetics of CN_urea_ and CN_urea_-**Ni(abt)2** are largely similar in pure water (Fig. S7†) and exhibit power law behavior. This similarity suggests that the probed decays in the absence of TEOA are dominated by recombination due to deeply trapped electrons which cannot transfer to co-catalysts, as previously reported, and that charges on the co-catalyst exhibit the same recombination behavior as those on the CN itself under these conditions. Upon addition of TEOA, the half time with respect to the signal at 10 μs almost doubles from CN_urea_ (100 μs) to CN_urea_-**Ni(abt)2** (190 μs) when probed at 700 nm (Fig. S7A†). In contrast, the decay profiles at 600 nm and 800 nm, *i.e.* outside the spectral region of the 700 nm peak, are largely identical for the two materials (Fig. S8†). Kinetic traces of **Ni(abt)2** were obtained from subtraction of CN_urea_ from CN_urea_-**Ni(abt)2** (Fig. S7B†), and then normalized at 10 μs. This allows for an estimation of the decay kinetic related to the covalently bound molecular catalyst itself (Fig. 6B). The significantly slower decay over the μs to ms timescale is in agreement with the timescales usually relevant for proton reduction.^30,31^ Furthermore, the drastically increased half time of the estimated co-catalyst kinetics (7 ms) compared with the kinetics of bare CN_urea_ (10 μs) obtained from Fig. 6B may be explained by a higher spatial separation of charges through the electron accepting molecular catalyst bound to the system. Overall these spectroscopic data indicate the sub-microsecond transfer of electrons from CN to states associated with the covalently bound **Ni(abt)2** catalyst, resulting in long lived catalyst reduction to subsequently drive proton reduction. In related reports with CN and molecular catalysts freely diffusing in solution the timescale of electron transfer was >2 s,^9^ thus tight binding of the catalyst to the CN offers six orders of magnitude higher rates of electron transfer compared to diffusion-limited examples.

## Conclusions

In conclusion we have presented a versatile strategy for covalently attaching well-defined molecular catalysts to CNs. We combine the low-cost and tunable nature of CN with the well-defined, tunable and selective nature of Ni-based molecular catalysts. This approach has led to a more than three times enhancement in TON compared with the highest reported so far. The system evolves H_2_ at an average rate of 70 μmol h^−1^ g^−1^ for 192 h. Time-resolved spectroscopy shows six orders of magnitude faster charge transfer to the covalently bound molecular catalyst compared to reports of co-catalysts in solution. The result is seven hundred times longer lived photoelectrons on the Ni catalyst than in bare CN. Doped CNs show activity with *λ* > 475 nm. This can pave the way for robust, efficient and easily-recycled photocatalysts in fields such as solar fuels, ammonia production and photoredox catalysis. Given the commercial availability of chlorinated ligands (bipyridines, phenanthrolines, terpyridines, porphyrins, phthalocyanines) this work is anticipated to provide a solid basis for future studies and discoveries of low cost, noble metal-free hybrid photocatalysts.

## Experimental section

### General methods and chemicals used

All starting materials were purchased from Aldrich unless mentioned otherwise. Dry toluene was purchased from Acros. Tetrabutylammonium bis(3,6-dichloro-1,2-benzenedithiolato)nickelate was purchased from TCI. Graphitic CNs, ONLH and FAT 1.0 were prepared according to reported procedures.^3–5^

#### Preparation of **Ni(abtCl)2**

A 50 mL round bottom flask was charged with 2 mL H_2_O and 8 mL EtOH. KOH (176 mg, 3.13 mmol) was added followed by 2-amino-4-chlorobenzenethiol (500 mg, 3.13 mmol). DMSO was added (2 mL) and the mixture was stirred and sonicated until the ligand had fully dissolved. NiCl_2_·6H_2_O (372 mg, 1.57 mmol) was dissolved in 2.5 M NH_3_ solution (10 mL) and added dropwise to the ligand solution to form a green/blue precipitate. The mixture was stirred for 5 h and then the solid was collected by filtration. The solid was ground to a powder and suspended in 30 mL H_2_O with 400 mg KOH and bubbled with air for 6 h. The solid was collected and washed once with water and once with CH_2_Cl_2_. Recrystallization from DMF/CHCl_3_ yielded pure **Ni(abtCl)2** (52 mg, 9%). Elem. anal. calcd: C, 38.55; H, 2.16, N, 7.49. Found: C, 38.34; H, 2.14; N, 7.16. ^1^H NMR DMSO-d_6_: 8.10 (bs) showing paramagnetic character.

#### CN – catalyst hybrid

In an air-free glovebox a pressure tube was charged with CN (100 mg), dry toluene (10 mL) and ^*t*^BuOK (6 mg). Nickel catalyst was then added (4 mg). The tube was sonicated for 1 h followed by heating at 135 °C for 36 h. The tube was allowed to cool and 10 mL water was added, followed by 30 mL DMSO. The solid was then separated by centrifugation (5000 rpm, 20 min). The solid was washed repeatedly with DMSO until the supernatant was colorless, followed by washing with water. The resulting CN – catalyst hybrid was dried at 70 °C overnight.

#### CN + absorbed catalyst

**Ni(abtCl)2** was dissolved in DMF (5 mL) and separately CN (250 mg) was suspended in DMF (5 mL). The **Ni(abtCl)2** solution was added dropwise to the CN suspension with sonication. The DMF was removed by rotary evaporation. The CN was then washed using an identical method as that for the hybrid material. The solid was dried at 70 °C overnight.

### General characterization methods and equipment

UV/Vis absorption spectra were recorded on an Agilent Technologies Cary 5000 UV/Vis spectrometer. X-ray photoelectron spectroscopy was performed on a Thermo Scientific XPS K-alpha machine using monochromatic Al-Kα radiation. Survey scans were collected in the range of 0–1350 eV (binding energy) at a pass energy of 200 eV. Higher resolution scans were recorded for the main core lines at a pass energy of 50 eV. The analysis was performed on Casa XPS software. Inductively coupled plasma atomic emission spectroscopy was performed on an Agilent Technologies 4210 MP-AES (calibrated using commercial standards; Aldrich 42242). Samples (∼40 mg) were stirred in methylsulfonic acid (400 μL) for several days then diluted to 4 mL and submitted to the ICP-AES. During photocatalysis, amounts of hydrogen in the gas phase were measured on a Varian 430 GC equipped with a molecular sieve column and TCD detector which was calibrated using known amounts of H_2_ gas. Specific surface area measurements were taken using the BET method (N_2_ absorption, TriStar 3000, Micromeritics).^3–5^

### Single crystal X-ray diffraction

X-ray diffraction data on a thin single crystal of **Ni(abtCl)2** at 150 K were obtained using an Agilent Oxford Diffraction SuperNova equipped with a microfocus Cu Kα X-ray source, an Atlas CCD detector, and a Cryojet5® cooler. A half-sphere of data were collected to 0.84 Å resolution with each 1° scan frame in *ω* collected twice; count time totaled 20 s for low 2*T* angle frames and 80 s for high angle ones with a total measurement time of *ca.* 20 hours. Data reduction to *hkl* and |*F*(*hkl*)2| was performed using CrysAlisPro (version 1.171.39.46 from Rigaku Oxford Diffraction^32^). The crystal structure was solved and refined by least-squares within the Olex2 program suite^33^ using the structure-solution program ShelXT^34^ and the refinement program ShelXL.^35^ The position of all hydrogen atoms could be seen in the difference Fourier map. The positions of all atoms were refined freely, with isotropic displacement parameters for the H atoms and with anisotropic displacement parameters for the remaining atoms.

### Light-driven hydrogen evolution studies

A glass photoreactor with a quartz window was charged with photocatalyst (10 mg) and a solution of H_2_O with 10% TEOA (50 mL). The reactor was sealed, sonicated and then purged with argon for 30 min. The suspension was irradiated with a 300 W Xenon light source (Newport 66485-300XF-R1) equipped with appropriate band-pass filters. For the quantum yield measurement, 30 mg of catalyst was used. The apparent quantum yield (AQY) was calculated by using the following formula:AQY = (2 × number of evolved hydrogen molecules)/(number of incident photons) × 100%

The light intensity measurements were taken by an optical power meter (Newport 1918 R) with an appropriate band pass filter (365, 400, 420 nm, *λ* ± 10 nm at 10% of peak height, Comar Optics) inserted between a 300 W Xe light source (Newport 66485-300XF-R1) and the reactor. The gas evolution rates were calculated from the average of five runs.

### Transient absorption spectroscopy (μs to ms timescale)

Transient absorption spectroscopy was performed using a home-built setup in diffuse reflectance geometry. A ND:YAG laser (OPOTEK Opolette 355 II, 7 ns pulse width) was used as the excitation source and the third harmonic output at 355 nm was transmitted to the sample through a light guide. During each measurement, an excitation power density of 500 μJ cm^−2^ was used at a laser frequency of 0.4 Hz. The probe light was produced by a 100 W Bentham IL1 quartz halogen lamp. Long pass filters and an IR filter (H_2_O, 5 cm path length) positioned between the lamp and the sample minimized short wavelength irradiation and heating of the sample during measurement. Diffuse reflectance from the sample was filtered through a monochromator (Oriel Cornerstone 130) before being collected by a Si photodiode (Hamamatsu S3071). The acquisition was triggered through a photodiode (Thorlab DET10A) which was exposed to laser scatter between the laser and the light guide. Data before 1.8 ms was amplified by custom electronics (Costronics) and acquired by an oscilloscope (Tektronix DPO 3012). Following data was acquired on a National Instrument DAQ card (NI USB-6251). Kinetic traces were typically obtained from the average of 128 laser pulses. The sample was prepared by suspending 5.56 mg mL^−1^ material in water and sonicating the samples for 17 h. Centrifugation (2000 rpm, 4 min), followed decanting of the fine suspension and further dilution with TEOA or water yielded the final suspensions. Prior to each measurement, the samples were kept under Ar atmosphere and stirred. Data was acquired using home-built software written in LabVIEW and further processed using home-built MATLAB tools and OriginPro.

## Conflicts of interest

There are no conflicts to declare.

## Supplementary Material

SC-011-D0SC02986F-s001
