# Supplementary material for: Covalent grafting of molecular catalysts on C3NxHy as robust, efficient and well-defined photocatalysts for solar fuel synthesis
Source: Chem Sci. 2020 Jul 24;11(32):8425–32. doi: 10.1039/d0sc02986f (PMC8163425; doi:10.1039/d0sc02986f)
Supplement: SC-011-D0SC02986F-s001 [file SC-011-D0SC02986F-s001.pdf]

Electronic Supplementary Material (ESI) for Chemical Science.
This journal is © The Royal Society of Chemistry 2020

Supporting Information for

Covalent grafting of molecular catalysts on $C_3N_xH_y$ as robust, efficient and well-defined photocatalysts for solar fuel synthesis

Christopher D. Windle,[†] Alexander Wieczorek,[‡] Lunqiao Xiong,[†] Michael Sachs,[‡] Carlota Bozal-Ginesta,[‡] Hy-ojung Cha,[‡] Jeremy K. Cockcroft,[§] James Durrant[‡] and Junwang Tang[*,†]

[†] Department of Chemical Engineering, UCL Torrington Place, London, WC1E 7JE, UK

[‡]Department of Chemistry and Centre for Plastic Electronics, Imperial College London, White City Campus, London, W12 0BZ, United Kingdom

[§]Department of Chemistry, University College London, 20 Gordon Street, London, WC1H 0AJ, UK.

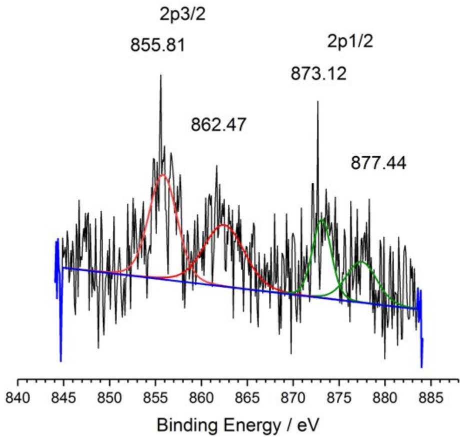

Figure S1. XPS of CN_{urea}-**Ni(abt)$_2$** after 24 h irradiation (λ > 420 nm, 10% TEOA, pH = 11)

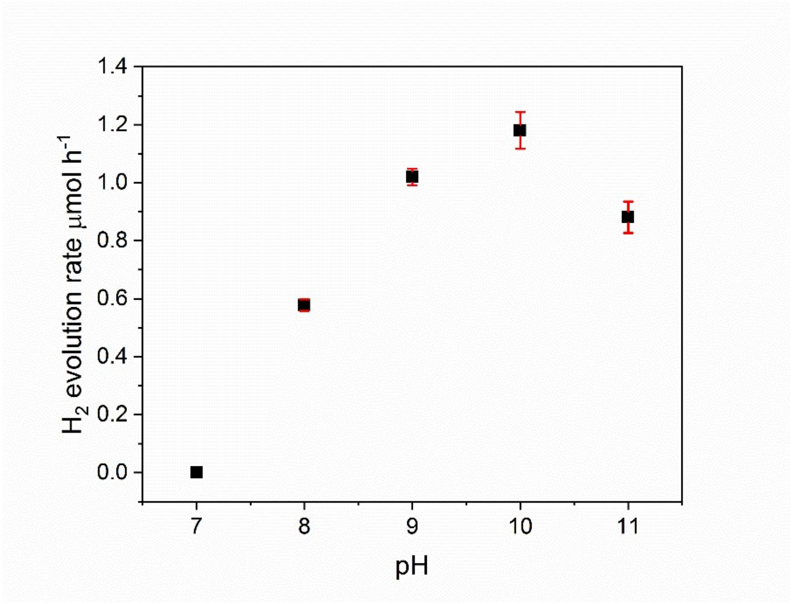

Figure S2. Effect of the pH of CN_{urea}-Ni(abt)$_2$ suspension on the rate of photocatalytic H_2 evolution in H_2O with 10% TEOA (pH = 11) and visible irradiation (λ > 420 nm) (adjusted by H_2SO_4).

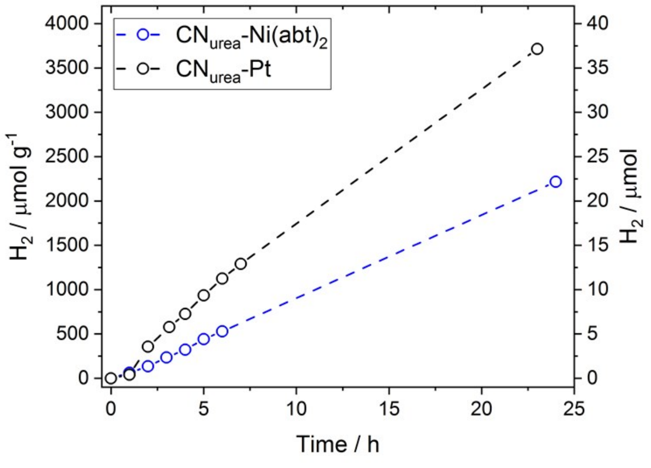

Figure S3. Photocatalytic hydrogen production by CN_{urea}-Ni(abt)$_2$ compared with CN_{urea} with Pt loaded. The H_2 evolution rate on CN_{urea}-Ni(abt)$_2$ is 60% of that on Pt-CN while the turnover frequency of CN_{Urea}-Ni(abt)$_2$ is 9.2 h^{-1}, is 6.9 h^{-1} on Pt loaded CN under the same condition. All in H_2O with 10% TEOA (pH = 11) and visible irradiation (λ > 420 nm).. Similar amount of Pt was loaded on CN_{urea} by photodeposition, using full-arc of a 300 W Xenon light source and methanol as sacrificial agent. TOFs are calculated by the equation below:

$$TOF = (moles\ of\ desired\ product\ formed)/(moles\ of\ active\ centers * reaction\ time)$$

Catalyst loading amounts for CN-Ni(abt)$_2$ and CN-Pt are 0.1 μmoles and 0.23 μmoles respectively.

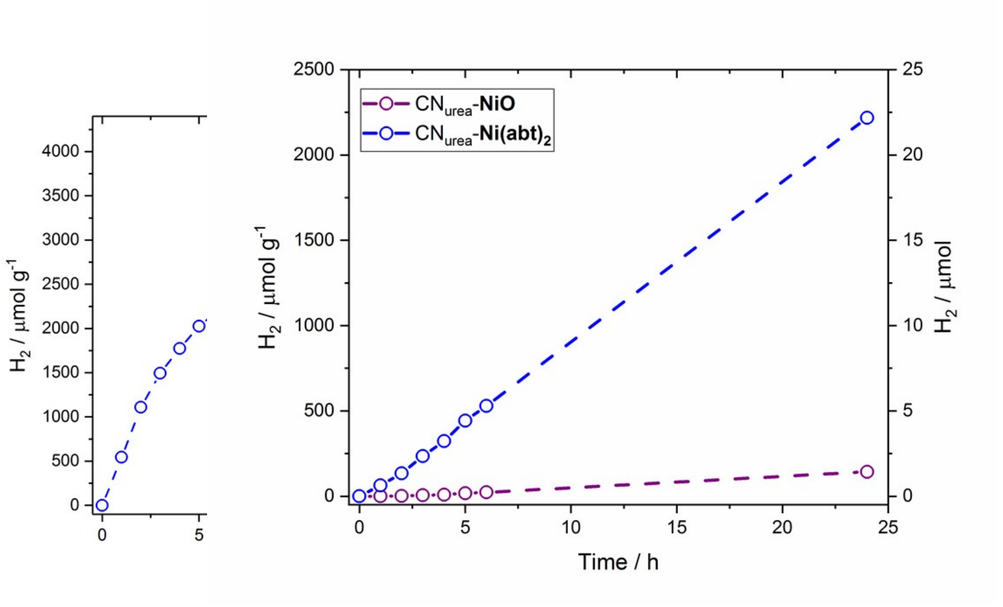

Figure S4. CN_{urea}-Ni(abt)$_2$ with λ > 320 nm

Figure S5. Photocatalytic hydrogen production by CN_{urea}-Ni(abt)$_2$ and CN_{urea} loaded with NiO in H_2O with 10% TEOA (pH = 11) and visible irradiation (λ > 420 nm).

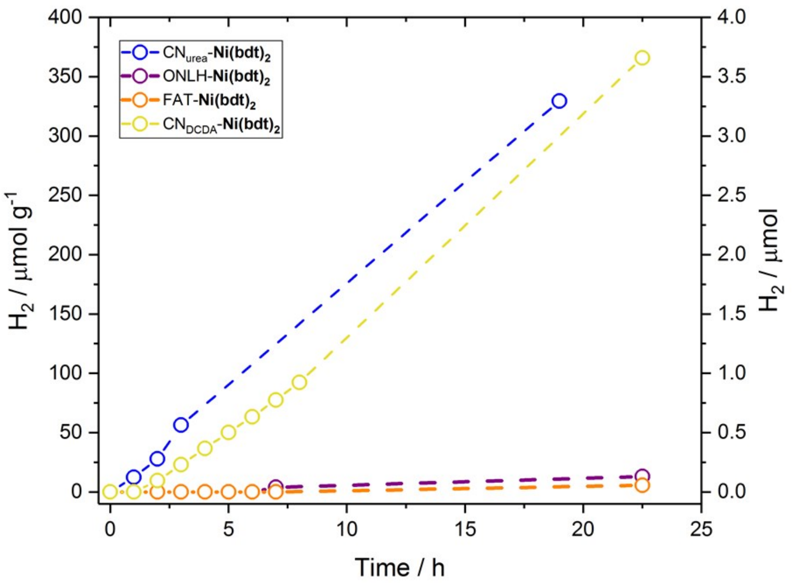

Figure S6. H_2 evolution of Ni(bdt)$_2$ hybrid materials (10% TEOA in H_2O, λ > 420 nm, pH = 11). Considering the estimated potentials of conduction bands, it is clear that CN_{urea} and CN_{DCDA} could be reduced to transfer electrons to both catalysts (Ni(abt)$_2$ and Ni(bdt)$_2$). For Ni(abt)$_2$, there is a significantly greater driving force for electron transfer from CN_{urea} and CN_{DCDA}. When the driving force is so sufficient, the degree of polymerization determines hydrogen-evolution rates from water. Higher polymerization degree in CN_{urea} resulted to better migration of charger carriers to active sites. Therefore, H_2 evolution rate for CN_{urea}-Ni(abt)$_2$ is significantly higher than that of CN_{DCDA}-Ni(abt)$_2$. In the case of Ni(bdt)$_2$, driving force is so moderate that the polymerization degree could not make a big difference. Hence the rates for CN_{urea}-Ni(abt)$_2$ and CN_{DCDA}-Ni(abt)$_2$ are similar.

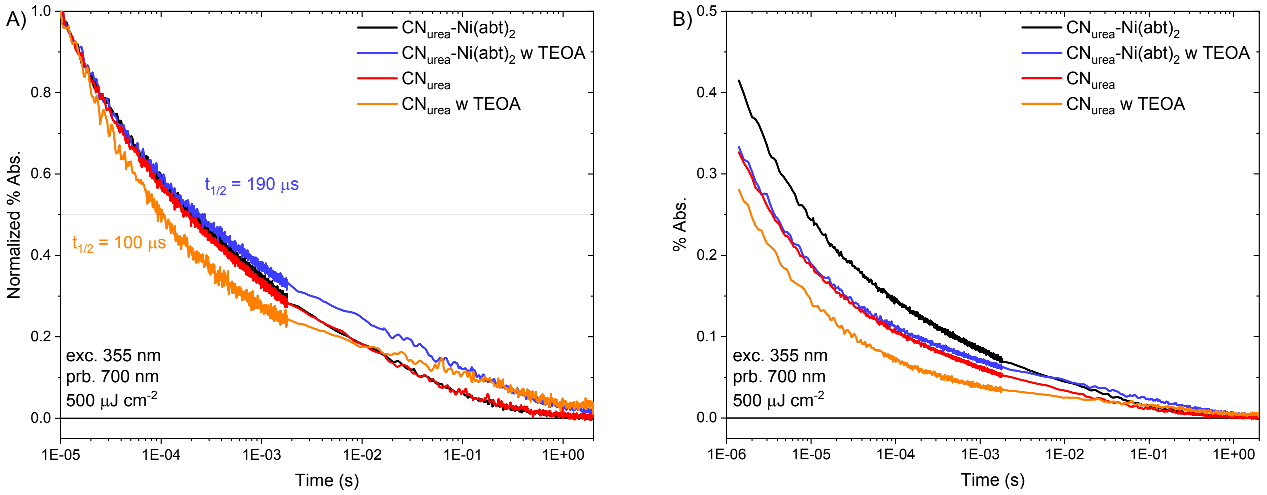

Figure S7. A) Normalized kinetics at a probe wavelength of 700 nm with half time ($t_{1/2}$) of decays in TEOA containing solvents. B) Raw traces at the same probe wavelength.

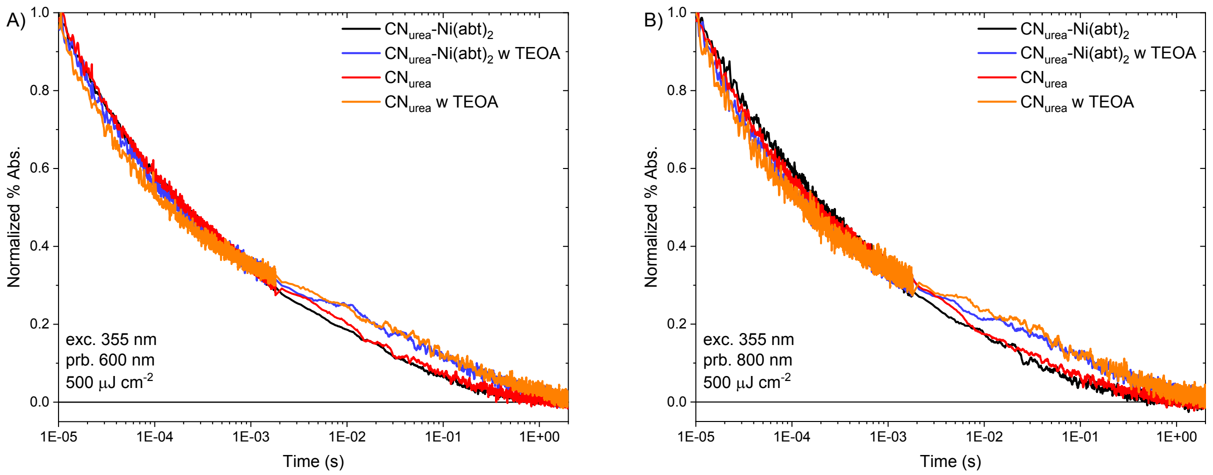

Figure S8. Normalized kinetics of investigated samples probed at A) 600 nm and B) 800 nm.

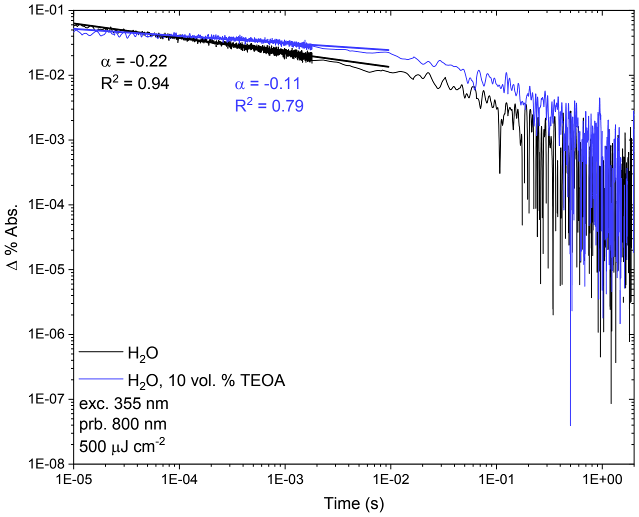

Figure S9. Fit parameter for an assumed power-law decay ($A \propto t^{-\alpha}$) of the estimated co-catalyst kinetics from CN_{urea} and CN_{urea}-**Ni(abt)$_2$** in both solvent conditions, probed at 700 nm.

Table S1. Crystal data and structure refinement for **$Ni(abtCl)_2$**

Identification code	exp_1401
Empirical formula	$C_{12}H_8Cl_2N_2NiS_2$
Formula weight	373.93
Temperature / K	150
Crystal system	monoclinic
Space group	*C*2/*c*
a / Å	29.8655(12)
b / Å	3.81961(14)
c / Å	11.3728(3)
α / °	90
β / °	96.147(3)
γ / °	90
Volume / Å^3	1289.88(8)
Z	4
ρ_{calc} / g cm^{-3}	1.926
μ / mm^{-1}	8.872
F(000)	752.0
Crystal size / mm^3	0.101 × 0.059 × 0.015
Radiation	Cu Kα (λ = 1.54184 Å)
2θ range for data collection / °	11.922 to 146.092
Index ranges	$-32 \leq h \leq 36, -4 \leq k \leq 4, -13 \leq l \leq 13$
Reflections collected	3847
Independent reflections	1267 [R_{int} = 0.0209, R_{sigma} = 0.0181]
Data/restraints/parameters	1267/0/104
Goodness-of-fit on F^2	1.132
Final *R* indexes [$I \geq 2\sigma(I)$]	R_1 = 0.0340, wR_2 = 0.0924
Final *R* indexes [all data]	R_1 = 0.0354, wR_2 = 0.0935
Largest diff. peak/hole / e Å^{-3}	0.40/−0.47

Table S2. Comparison of carbon nitride – molecular catalyst systems for H_2 evolution.

Carbon Nitride Precursor	Catalyst	Solvent	Light source	Running time / h	H_2 rate / µmol h^{-1}	TON	TOF / h^{-1}	QY	Ref DOI
Cyanamide	NiP	H_2O	λ > 300 nm	3	2.2	170	109	0.37% (365 nm)<br>0.04% (460 nm)	10.1002/anie.201406811
			λ > 420 nm	4	0.4	60	15		
Urea	Cobaloxime-pyrene	CH_3CN-H_2O 1/9	λ > 400 nm<br>300 W Xe	9	20	281	40		10.1039/c4ra01413h
Dicyandiamide	Ni TEOA complex	H_2O	λ > 400 nm<br>500 W Xe	60	7	281	10	1.5% (400 nm)	10.1002/cssc.201200490
Melamine then KSCN	NiP	H_2O	λ > 400 nm<br>100 mW cm^{-2}	24	0.8	200	12	15% (360 nm)	10.1021/jacs.6b04325
			AM 1.5	24	1.5	425	31		
Melamine then KSCN then graphene oxide	NiP	H_2O	AM 1.5	4	6	360	116		10.1021/acscatal.8b01969
Melamine	Cobaloxime	H_2O	350 - 740 nm<br>300 W Xe	8	2.6	4	0.5	0.62 (365 nm)	10.1039/c3cp53350f
Urea	$Ni(abt)_2$	H_2O	λ > 420 nm<br>300 W Xe	192	1.2	1360	9.2	~2.3% (365 nm)<br>~1.5% (400 nm)<br>0.7% (420 nm)	This work
			λ > 320 nm<br>300 W Xe	24	5.7	422	54.3		
Semicarbazide hydrochloride	$Ni(abt)_2$	H_2O	λ > 475 nm<br>300 W Xe	24	0.2	22	1.2		This work

Table S3. Comparison of H_2 evolution rate for different carbon nitride – molecular catalysts in this work

**Catalyst**	**H_2 rate / µmol h^{-1}**
CN_{DCDA}-**Ni(abt)$_2$**	0.14
FAT-**Ni(abt)$_2$**	0.28
ONLH-**Ni(abt)$_2$**	0.34
CN_{urea}-**Ni(abt)$_2$**	0.92
CN_{urea}-**Pt**	1.55
CN_{DCDA}-**Ni(bdt)$_2$**	0.16
FAT-**Ni(bdt)$_2$**	0.002
ONLH-**Ni(bdt)$_2$**	0.005
CN_{urea}-**Ni(bdt)$_2$**	0.17

Reaction condition: 10 mg catalyst in a solution of H_2O with 10% TEOA (50 mL) irradiated with a 300 W Xenon light source (Newport 66485-300XF-R1) equipped with a λ > 420 nm filters for 24 h.
